# Surface Glass Fiber Hybridization for Enhanced Low-Velocity Impact Resistance in CFRP T-Stiffened Panels

**DOI:** 10.3390/polym18101259

**Published:** 2026-05-21

**Authors:** Yuhuan Yuan, Yangsheng Gao, Debin Song, Wei Xi, Jia Huang, Jiali Tang

**Affiliations:** 1 School of Civil Aviation, Northwestern Polytechnical University, Xi’an 710072, China; 2 Composites Center, COMAC Shanghai Aircraft Manufacturing Co., Ltd., Shanghai 201324, China; 3 School of Aeronautics, Northwestern Polytechnical University, Xi’an 710072, China

**Keywords:** carbon-fiber-reinforced polymer, glass-fiber-reinforced polymer, hybrid composites, T-stiffened panels, low-velocity impact, cohesive zone model

## Abstract

This study systematically investigates the low-velocity impact response of aerospace-grade carbon-fiber-reinforced polymer (CFRP) T-stiffened panels. Through drop-weight impact tests at 20 J and 35 J energies and Cohesive Zone Model (CZM) numerical simulations, a comparative analysis was performed on two composite configurations: the pure CFRP baseline (Configuration A) and the hybrid configuration incorporating surface glass fiber layers (Configuration B). High-fidelity correlation between experimental and numerical results was achieved, validating the progressive damage evolution of the matrix and fiber constituents. The main findings demonstrate that the hybrid Configuration B exhibits significantly superior impact resistance compared to the monolithic CFRP Configuration A. The introduction of surface glass fiber layers produces a synergistic hybrid effect in the composite system. This surface layer acts as a protective buffer, effectively attenuating the impact load before it propagates to the underlying carbon fiber laminate. As a result, the hybrid structure absorbs more energy and effectively suppresses rapid crack propagation. Under 35 J impact energy, Configuration B avoids the brittle failure of the matrix observed in Configuration A, achieving a 24% increase in permanent energy absorption. This surface hybridization strategy provides an effective method for improving damage tolerance and preserving the structural integrity of advanced composite stiffened panels.

## 1. Introduction

The primary stiffener configurations for carbon-fiber-reinforced polymer (CFRP) stiffened panels are classified by cross-sectional geometry into four fundamental types: T-shaped, I-shaped, Hat-shaped, and J-shaped/L-shaped [1,2]. Carbon-fiber-reinforced polymer stiffened panels perform critical structural functions in aerospace, wind energy, and automotive industries. Within commercial aviation, Hat-section stiffeners constitute primary wing spars and fuselage frames in Boeing 787 and Airbus A350 aircraft, where their exceptional buckling resistance sustains extreme aerodynamic loads while achieving 25–30% weight reduction versus metallic structures [3]. T-stiffeners reinforce secondary components such as wing skins and bulkheads, with their manufacturing adaptability accommodating complex contour formation requirements.

CFRP stiffened panels are highly susceptible to low-velocity impact (LVI) events in the 5–50 J energy range during manufacturing, maintenance, and service operations [4]. Typical scenarios include tool drops during assembly, equipment collisions in hangar environments, hail strikes at 20–30 m/s velocities, and debris impacts on runways [5]. These impacts often produce surface indentations shallower than 0.3 mm, resulting in Barely Visible Impact Damage (BVID) that evades visual detection under standard inspection protocols. The resulting internal damage manifests as multi-scale failure modes. Interlaminar delamination accounts for more than 80% of the total damage area and propagates along ply interfaces due to transverse shear stresses. Skin-stiffener interfacial debonding concentrates at curvature zones (such as hat-section radii) where peel stresses are highest. Matrix cracking initiates perpendicular to the fiber directions, while localized fiber fracture occurs beneath the impactor contact zone. This damage configuration significantly degrades structural integrity, reducing compression-after-impact strength by 40–60% and shear stiffness by over 30% through delamination-induced buckling mechanisms [6]. Under cyclic loading combined with hygrothermal exposure, delamination propagation rates accelerate 300–500%, causing further strength reductions to 55% of initial values after 10^4^ load cycles [7]. Documented failures include wing root delamination in A350 fleets from 28 J maintenance impacts and wind turbine blade fractures due to 35 J hail strikes, both triggering failures below certified limit loads [8]. These safety implications necessitate advanced mitigation strategies. Examples include nano-toughened resins that improve fracture toughness by 50%, embedded carbon nanotube sensor networks that enable real-time damage detection, and AI-driven digital twins that reduce CAI prediction errors to below 12%. Such approaches help ensure compliance with FAA 25.571 airworthiness mandates, which require 150% residual load capacity in the presence of BVID [9,10].

Experimental methodologies for fiber-reinforced laminates adhere to ASTM D7136 drop-weight protocols using hemispherical impactors at 5–30 J energies, with damage quantified through ultrasonic C-scanning and dent-depth measurements, followed by residual-strength evaluation via compression-after-impact (CAI) per ASTM D7137. For stiffened panels, tests target skin-stiffener junctions and inter-stiffener bays using aerospace-representative geometries under constrained boundary conditions [11,12,13,14].

Hybridization represents an effective strategy for improving the impact performance of composite laminates. Glass fibers, characterized by their high elongation at break and superior toughness, offer complementary properties to carbon fibers, which exhibit high strength and modulus but relatively low failure strain. The synergistic combination of these two fiber types in hybrid laminates has demonstrated promising potential for enhanced impact performance. This behaviour has been experimentally confirmed by Chen et al. [15], who investigated interlayer carbon/glass hybrid laminates and reported that hybrid configurations containing both carbon and glass fibre layers exhibited significantly improved low-velocity impact resistance compared with pure carbon fibre laminates. The enhancement in impact performance was attributed to the combined damage mechanisms of the two constituents, where brittle fracture and fibre breakage dominated in carbon-rich regions, while glass fibre layers contributed to increased deformation capacity, energy dissipation, and crack deflection during impact loading. This synergistic interaction results in a more progressive failure process and delays catastrophic structural collapse. Zhang et al. [16] investigated the low-velocity impact behavior of carbon/glass hybrid composites and demonstrated that hybrid laminates exhibited improved impact resistance and energy absorption capability compared with pure carbon fiber composites. The impact response was closely related to the hybrid architecture and the deformation characteristics of carbon and glass fibers. Regarding the low-velocity impact response of carbon/glass hybrid laminates, existing investigations have primarily focused on the influence of stacking configurations. For marine composite structures, laminates with (C2G2) S stacking sequence demonstrated optimal low-velocity impact performance, achieving up to 34% higher energy absorption compared to alternative hybrid configurations [17]. Previous studies further revealed that hybridization between carbon and glass fibers could effectively tailor impact damage evolution and energy dissipation mechanisms. Hybrid laminates generally exhibited improved damage tolerance under low-velocity impact, although higher impact energies could still induce severe delamination and matrix cracking [18,19,20].

Delamination constitutes the most critical damage mode in laminated composites subjected to low-velocity impact. The extent and propagation behavior of delamination directly determine the residual load-bearing capacity of impacted structures. Numerical investigations incorporating multiple damage modes have elucidated the complex interplay between matrix cracking, fiber fracture, and interfacial delamination in carbon/glass hybrid laminates under impact loading [21,22,23,24,25,26,27,28,29,30,31,32,33]. The introduction of glass fibers modifies the interlaminar stress distribution and potentially inhibits delamination propagation through their enhanced fracture toughness. However, existing research has predominantly concentrated on bulk hybridization effects achieved through alternating carbon and glass plies throughout the laminate thickness. Limited attention has been devoted to the specific localized hybridization configuration involving glass fiber layers confined to the surface regions. The surface, being the direct interface for impact contact, plays a decisive role in damage initiation and subsequent evolution. Surface glass fiber layers may buffer impact contact forces through their enhanced deformability and modify stress wave propagation characteristics, thereby influencing delamination behavior within the underlying carbon fiber-dominated plies. To date, the suppression effect of surface glass fiber layers on delamination damage in carbon fiber primary load-bearing laminates remains inadequately documented, and the underlying mechanisms warrant further elucidation [34,35,36,37,38,39,40,41].

In light of the aforementioned research gaps, the present study investigates the low-velocity impact behavior of aerospace-grade T800 carbon-fiber-reinforced polymer laminates modified by incorporating glass fiber layers on the external surfaces. Low-velocity impact experiments coupled with finite element simulations are conducted to systematically compare and analyze the impact response, damage morphology, and delamination area of laminates with and without surface glass fiber layers. The mechanisms underlying delamination suppression attributable to surface glass fiber layers are elucidated, aiming to provide theoretical foundations and experimental support for the impact-resistant design optimization of advanced aerospace composite structures.

## 2. Specimens and Experiment

### 2.1. Materials and Specimen Fabrication

The composite material system utilized in this study consists of T800-grade carbon-fiber reinforcement integrated with an epoxy resin matrix, achieving a nominal fiber volume fraction of 66%. Configuration A serves as the baseline, manufactured using a co-bonding process. Configuration B is also fabricated via co-bonding but incorporates localized surface hybridization, where 1080-grade glass fiber fabric layers are applied to both the front and back surfaces of the vertical stiffener-web. These glass fiber layers are oriented at a 0° ply angle with a thickness of 0.05 mm per layer; the detailed stacking sequences are summarized in Table 1.

To ensure structural consolidation and optimal matrix properties, strict manufacturing parameters were implemented. The thermal curing cycle featured an intermediate dwell platform at 135 °C for 140~180 min, followed by a final curing stage at 180 °C for 240~300 min. Furthermore, for components involving thermal compaction (hot debulking) during the manufacturing process, stringent temperature-time constraints were enforced to prevent premature resin advancement. Specifically, the cumulative duration within the 26~43 °C temperature range was restricted to a maximum of 24 h, while the cumulative time within the 43~90 °C range was strictly limited to no more than 150 min.

### 2.2. Experimental Set-Up

**Figure 1:**
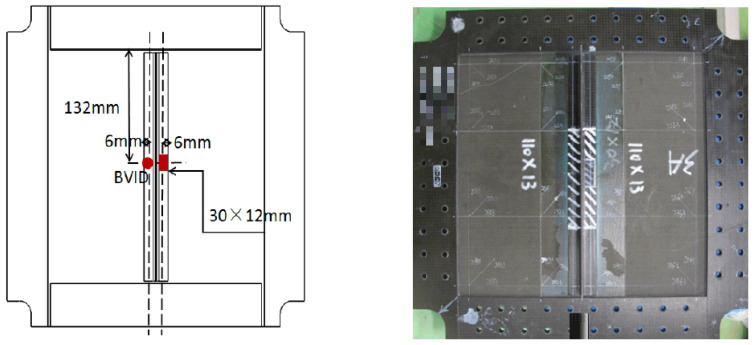
Schematic diagrams and photographs of the two configurations.

The schematic representations of the specimens corresponding to the two configurations, designated as Configuration A and Configuration B, are presented in Figure 1. The specimens used in this study were T-shaped stiffened composite panels, consisting of a flat skin and an integrally bonded vertical stiffener. The geometry corresponds to a typical T-joint configuration widely adopted in aerospace structures. The skin is a rectangular laminate plate, while the stiffener comprises a vertical web and a horizontal flange, forming a T-shaped cross-section. The web is oriented normally to the skin surface, and the flange is co-cured or co-bonded to the skin, providing structural continuity.

The in-plane dimensions of the specimens exceeded the central cut-out of the supporting fixture (120 mm × 200 mm), such that the unsupported impact region was fully contained within the specimen interior. The impact was applied at the geometric center of the exposed skin area adjacent to the stiffener. The geometric proportions of the stiffener, including the web height and flange width, were selected in accordance with representative aerospace configurations.

The specimen size also ensured sufficient distance between the impact location and the clamped boundaries, thereby reducing boundary-induced constraints and enabling an accurate characterization of impact-induced damage in the stiffener–skin interaction region.

**Figure 2:**
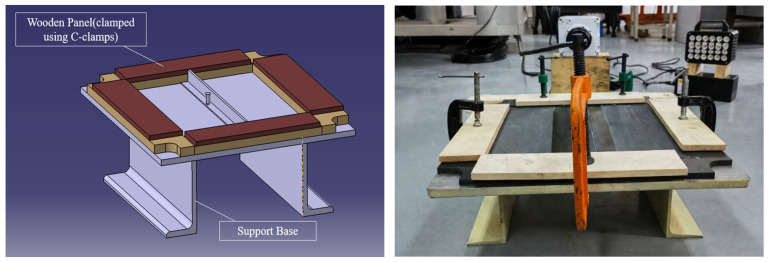
Schematic and photograph of the experimental setup.

As illustrated in Figure 2, the experimental setup employed a drop-weight impact system to evaluate CFRP T-stiffened panels under controlled low-velocity impacts. A 5 kg steel impactor with a 16 mm hemispherical tip was dropped from calibrated heights to deliver 35 J impacts (3.74 m/s) and 20 J impacts (2.828 m/s). Specimens were clamped using metallic fixtures with a consistent tightening torque to ensure repeatable boundary conditions. The specimens were firmly clamped on all four sides using metallic pressure strips and engineering clamps mounted on a rigid base. A consistent preload was applied to ensure repeatable and uniform boundary conditions. Each test condition was repeated at least three times to ensure statistical reliability.

**Figure 3:**
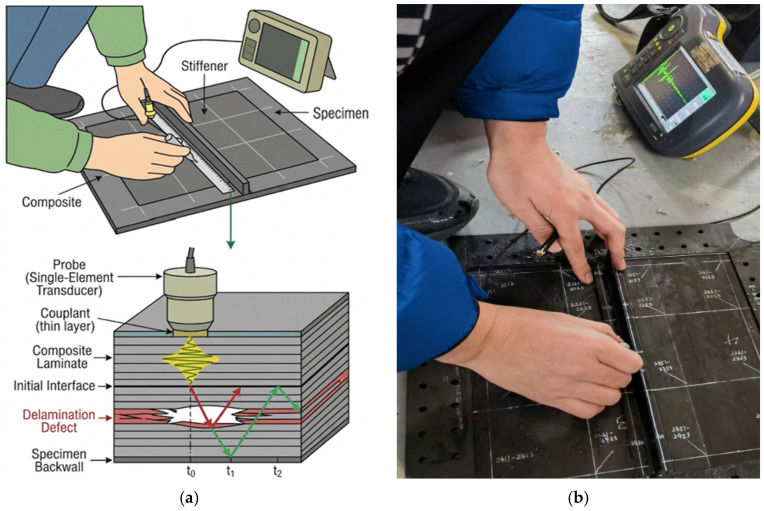
Ultrasonic A-scan inspection: (**a**) schematic diagram of signal response from a delaminated composite laminate; (**b**) photograph of the portable ultrasonic testing system and specimen during inspection.

Ultrasonic A-scan inspection was employed to characterize the internal damage of the impacted specimens, as illustrated in Figure 3. A portable ultrasonic testing system equipped with a single-element transducer was used in this study. The probe was connected to the data acquisition unit and operated in pulse–echo mode.

Prior to testing, a coupling medium was applied to the specimen surface to ensure effective transmission of ultrasonic waves. The probe was then manually positioned at specific locations across the surface following a predefined grid path, covering the entire region of interest, including the stiffener and adjacent skin areas.

During the inspection process, the time-of-flight (TOF) and amplitude of the reflected ultrasonic signals (A-scans) were continuously monitored. Internal defects, such as delamination, were identified by observing intermediate echoes between the initial pulse and the back-wall reflection, or by detecting a significant attenuation of the back-wall signal. The data collected from these discrete A-scan points were subsequently used to map and quantitatively analyze the damaged area.

## 3. Numerical Simulation

### 3.1. Cohesive Zone Model (CZM)

The Cohesive Zone Model (CZM) is a computational approach for simulating interfacial failure through a traction-separation law that governs progressive damage evolution. Based on Barenblatt–Dugdale theory, it eliminates stress singularities by introducing cohesive stresses in the fracture process zone that vary with displacement jumps. The model characterizes interface behavior through stiffness properties, damage initiation criteria, and energy-based softening laws, enabling accurate prediction of mixed-mode delamination in composites and bonded joints. Parameter calibration typically requires experimental data from standardized fracture tests, with careful attention to mesh sensitivity for numerical convergence [42].

For single type I and type II cracks, the bilinear cohesive model is defined by the delamination initiation point ($\delta^{0}$, $\sigma^{0}$) and the complete failure point ($\delta^{c\;}$, 0). The value of separation at damage initiation is expressed as:

(1) $${\delta^{0}}_{i}=\frac{{\sigma^{0}}_{i}}{E}$$

where i represents mode I or II cracking; ${\delta^{0}}_{i}$ denotes the value of separation at damage initiation; ${\sigma^{0}}_{i}$ is the stress at damage initiation; and *E* symbolizes the stiffness of cohesive elements. According to the triangular shape of the bilinear cohesion constitutive, the value of separation (${\delta^{c}}_{i}$) at the complete failure of the interface can be defined by

(2) $${\delta^{c}}_{i}=\frac{2G_{ic}}{{\sigma^{0}}_{i}}$$

where $G_{ic}$ is the fracture toughness of the interface, which is defined as the enclosed area under the traction–separation curve.

**Figure 4:**
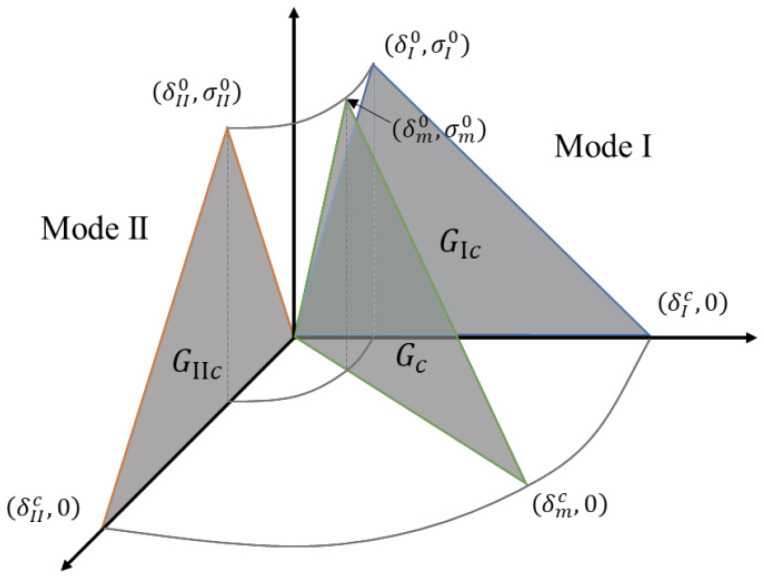
Schematic diagram of mixed-mode cohesive zone model.

Figure 4 presents the schematic representation of the mixed-mode cohesive zone model, illustrating the constitutive relationship between traction and separation displacement under combined loading conditions.

### 3.2. Finite Element Model (FEM)

**Figure 5:**
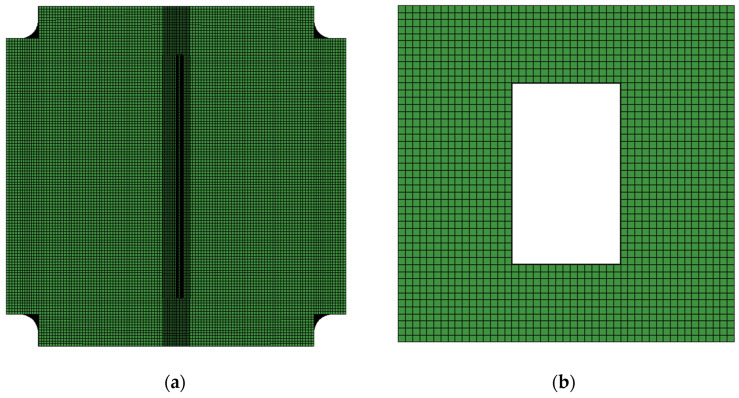

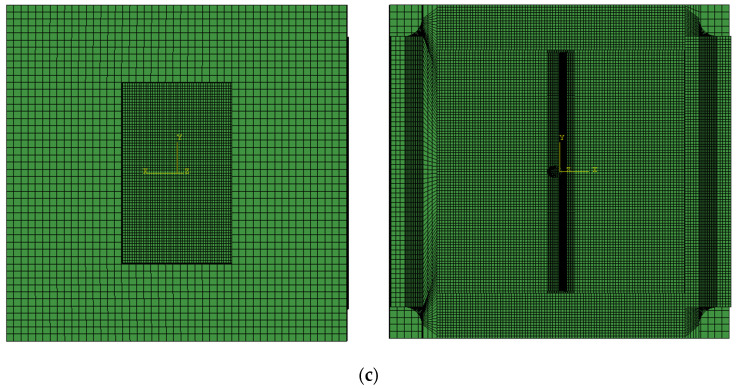
Numerical simulation diagram of the model: (**a**) composite panel; (**b**) test fixture; (**c**) structural assembly.

Figure 5 presents the primary focus of meshing on the discretization of the composite laminate. A nominal element size of approximately 3 mm was adopted for the composite panel, with thickness-direction layering structured according to the ply configuration: 14 layers for the skin, 9 layers for the bottom flange, and 8 layers for the web, which corresponds to Configuration A. To simulate delamination between the flange and web, minimal-thickness cohesive elements were embedded between adjacent solid plies. The final mesh comprised a total of 330,000 elements for this component. The remaining parts were meshed with reasonable coarseness, exhibiting negligible influence on computational results. The support platform was hollowed at the center as specified, with a cutout dimension of 120 × 200 mm.

For Configuration B, particular attention was given to damage progression in the T-stiffened panel. Consequently, the skin mesh was simplified using 3 × 8 mm rectangular elements, while the T-stiffener maintained a 3 mm element size. The thickness-direction layering consisted of 14 plies for the skin and 8 plies each for the flange and web. Zero-thickness cohesive elements were similarly implemented for interlaminar failure modeling, yielding a total of 201,576 elements for this configuration.

The material and interfacial parameters used in this study were calibrated based on experimental measurements and are consistent with the authoritative dataset reported by Dong and Davies (2012) [43]. This calibration approach ensures the physical relevance and reliability of the numerical model. As a seminal study in the field of carbon/glass hybrid composites, their work provides a comprehensive and validated dataset on T800-grade carbon fiber and glass fiber reinforcements in an epoxy matrix. By adopting these widely recognized parameters, the high fidelity of the current finite element model is ensured, allowing for an accurate prediction of damage initiation and propagation in both Configuration A and Configuration B.

Additionally, the impact hammer is made of steel with an elastic modulus of 2.1 × 10^5^ MPa and a Poisson’s ratio of 0.3, having a mass of 5 kg. The clamping fixture consists of wooden blocks characterized by an elastic modulus of 15,200 MPa and Poisson’s ratio of 0.29. The entire impact process was simulated using explicit dynamic analysis. The contact interaction was defined with hard normal behavior and tangential penalty friction (coefficient of 0.3), employing a general contact algorithm. Complete fixed constraints were applied to the top surface of the clamping fixture and the bottom surface of the support platform. The impact hammer was constrained in all degrees of freedom except the velocity direction, with an initial velocity of 3.741 m/s, resulting in calculated impact energies of 35 J.

## 4. Results and Discussion

### 4.1. Experimental Results and Analysis

Table 2 summarizes the post-impact damage dimensions and surface morphologies of both Configuration A and Configuration B under impact energies of 20 J and 35 J. A comparative analysis reveals that while damage areas in both configurations expand as the impact energy increases, Configuration B consistently exhibits significantly smaller damage footprints than Configuration A at identical energy levels.

When the energy escalates to 35 J, the protective effect of the hybrid architecture remains evident. Although the damage becomes more pronounced, Configuration B limits the web damage to 33 × 27 mm, which is a 17.5% reduction in width compared to the 40 × 27 mm observed in Configuration A.

Specifically, under the 20 J impact, Configuration A suffers extensive delamination with a flange-damage area of 60 × 8 mm and a web-damage area of 45 × 26 mm. In contrast, the hybrid Configuration B demonstrates superior damage resistance; its flange damage is significantly contained, reaching a minimum localized area of 7 × 8 mm, while the web damage area is reduced to 38 × 27 mm. Notably, the T-joint web of Configuration B exhibited no obvious visual delamination at this energy level, underscoring the effectiveness of the surface glass fiber reinforcements in suppressing crack initiation.

In the baseline Configuration A, cracks generated by the impact tend to propagate aggressively and laterally across the uniform carbon-fiber interface, resulting in a larger damage area. In contrast, the cracks in Configuration B encounter heterogeneous interfaces with mismatched moduli due to the presence of both glass and carbon fibers. This forces cracks within the matrix to deflect or branch, which increases the energy required to create new fractured surfaces. Furthermore, the glass fibers act as bridges across the composite layers to limit interlaminar displacement, significantly reducing the projected damage area at both 20 J and 35 J energy levels.

In this study, to accurately quantify the extent of interlaminar failure, the Elliptical Approximation Method was employed to calculate the projected damage area. Considering that impact-induced delamination in polymer composites typically manifests as an irregular elliptical or peanut-like shape rather than a perfect rectangle, the maximum length (*L*) and width (*W*) measured from the specimens were used as the major and minor axes, respectively. This approach provides a more realistic representation of the energy-dissipation zone compared to the conventional rectangular envelope method, effectively filtering out non-damaged corner regions and ensuring a high degree of fidelity in the subsequent comparative analysis between Configuration A and Configuration B.


(3) $$A_{delam}=\frac{\pi }{4}\times L\times W\approx 0.785\times L\times W$$

$A_{delam}$—The effective damage area

*L*—The maximum length

*W*—The maximum width

As shown in Table 3, a quantitative assessment of the delamination damage reveals that the percentage of damage reduction in Configuration B fluctuates with increasing impact energy. At 20 J, Configuration B demonstrates the most significant protective effect (37.81% reduction), suggesting that the surface glass fiber layers effectively disperse the impact load and suppress the expansion of interlaminar delamination during the early stages of low-energy impact. Under a higher energy impact of 35 J, although the absolute damage area increases, Configuration B still yields a nearly 20% performance improvement over Configuration A. This trend demonstrates that the hybrid design provides sustained structural integrity protection across varying load intensities.

### 4.2. Validation of the Numerical Model Against Experimental Results

**Figure 6:**
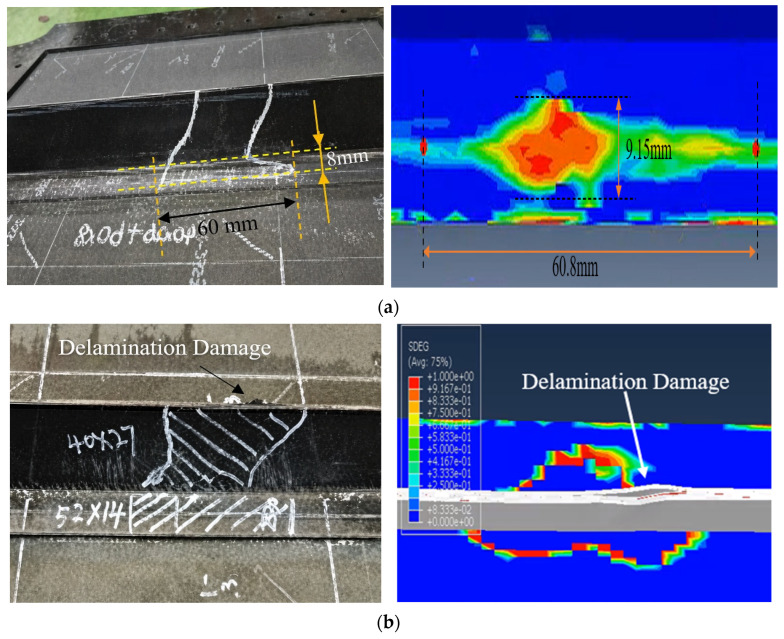
Comparative analysis of delamination damage in Configuration A under 20 J and 35 J Impact: (**a**) Configuration A under 20 J impact; (**b**) Configuration A under 35 J impact.

As illustrated in Figure 6, under an impact energy of 20 J, the delamination region significantly encompasses both the web and the bottom flange. For the bottom flange, the simulated damage area is 60.8 mm × 9.15 mm, which is in close proximity to the experimental measurement of 60 mm × 8 mm, yielding a marginal area deviation of 15.9%. Under the 35 J impact scenario, a detailed inspection of the localized damage confirms that the delamination within the T-joint web of Configuration A occurs at a location precisely predicted by the numerical model. This exceptional agreement is clearly visualized in Figure 6, where the simulated high-interlaminar-stress zone (red contour) in the web correlates perfectly with the actual fracture-initiating point on the experimental specimen. This remarkable positional correlation, combined with the similarity in crack-opening morphology, provides robust evidence for the high fidelity of the present finite element framework.

### 4.3. Comparative Simulation Analysis of Configuration A and Configuration B

**Figure 7:**
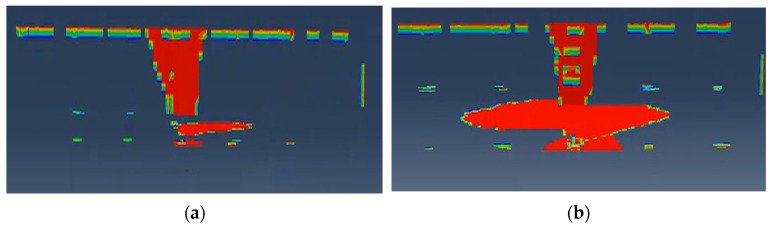

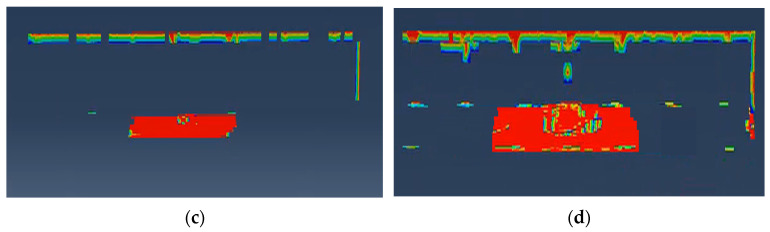
Comparison of damage areas for the two composite configurations: (**a**) Configuration A under 20 J impact; (**b**) Configuration A under 35 J impact; (**c**) Configuration B under 20 J impact; (**d**) Configuration B under 35 J impact.

The visual representation of impact damage in Figure 7 reveals a significant disparity between the two configurations. At a low-velocity impact energy of 20 J, the hybrid Configuration B demonstrates a remarkable suppression of delamination within the T-joint web compared to the monolithic Configuration A. In Configuration A, the impact-induced stress waves propagate vertically through the laminate thickness, leading to extensive interlaminar shearing in the brittle carbon-fiber web. Conversely, the surface glass fiber layer in Configuration B acts as a strategic buffer, promoting lateral energy dissipation within the skin and effectively shielding the critical web-to-skin interface from premature failure.

However, as the impact energy increases to 35 J, a discernible expansion in the web damage area is observed for the hybrid Configuration B. This phenomenon can be attributed to the energy-absorption threshold of the sacrificial glass fiber layers being surpassed. Once the surface hybrid architecture reaches its maximum strain capacity, the residual impact energy penetrates deeper into the underlying matrix and the T-joint core, causing the delamination to propagate into the vertical polymer web. Despite this growth, the total damaged volume in Configuration B remains more contained than in the pure CFRP system, validating that the hybrid-polymer approach successfully delays the onset of catastrophic structural dissociation by redistributing mechanical loads across heterogeneous polymer-fiber interfaces.

The experimental and numerical results can be jointly interpreted from the perspective of composite damage mechanics. The experimentally observed reduction in delamination area for Configuration B is consistent with the numerical prediction of a smaller interlaminar damage zone, indicating that the surface glass fiber layers effectively alter the stress transfer path and suppress delamination propagation.

From a micromechanical viewpoint, the distinct behavior between the two configurations is primarily governed by the mismatch in stiffness and failure strain between carbon and glass fibers. Carbon fibers, characterized by high modulus and low strain-to-failure, tend to exhibit brittle fracture under impact loading, leading to rapid crack initiation and unstable delamination growth. In contrast, glass fibers possess higher failure strain and fracture toughness, enabling greater deformation and energy dissipation.

This material heterogeneity directly explains the experimental observations. In Configuration A, the relatively uniform carbon fiber system facilitates straight crack propagation and extensive delamination, as confirmed by the larger damage areas measured experimentally. In Configuration B, the introduction of surface glass fiber layers modifies the stress distribution and promotes crack deflection and branching, thereby reducing the driving force for delamination growth.

The numerical results further support these mechanisms. The cohesive zone model captures the reduction in interlaminar damage and the more progressive failure process in Configuration B, which is consistent with experimentally observed suppression of delamination and increased energy absorption. The good agreement between experimental measurements and numerical predictions indicates that the model successfully reproduces the underlying damage mechanisms governed by fiber hybridization.

Overall, the combined experimental and numerical evidence demonstrates that the enhanced impact resistance of the hybrid configuration originates from the synergistic interaction between fiber constituents, which alters stress wave propagation, delays crack initiation, and suppresses delamination evolution.

As shown in Table 4, a comparative analysis between the experimental results and numerical predictions reveals a quantitative discrepancy ranging from approximately 10% to 20%. Specifically, the maximum error in the projected damage area is observed to be 20.05% at 20 J for Configuration B. Such a deviation is considered to be within an acceptable and normal range for studies involving low-velocity impact delamination in composite structures. This margin of error primarily stems from the inherent stochastic nature of material defects in fabricated specimens and the idealization of interlaminar properties within the cohesive zone model. Despite these minor discrepancies, the finite element model successfully captures the overall damage morphology and the trend of area reduction, thereby validating the reliability of the numerical framework for predicting the impact response of hybrid T-joint architectures.

### 4.4. Load–Displacement Curves and Energy Absorption Curves

The load–displacement and energy absorption responses were analyzed to elucidate the progressive damage process of the two configurations under 35 J impact. These curves illustrate the progressive damage evolution and internal mechanical response of the composite material under impact loading.

**Figure 8:**
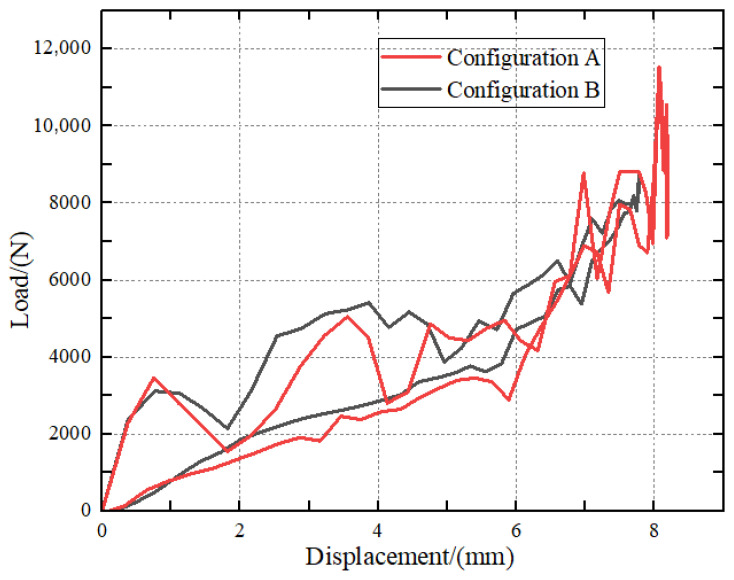
Curves for Configuration A and hybrid composite Configuration B.

The reference point at the leading edge of the impactor is selected to extract the load–displacement profiles, and the resulting curves for Configuration A and Configuration B under a 35 J impact are illustrated in Figure 8. Both configurations exhibit a similar initial bending stiffness, as indicated by the overlapping linear-elastic regions. However, Configuration A shows a significant load drop at approximately 3500 N, followed by intense oscillations, signifying brittle failure and rapid delamination propagation within the carbon fiber layers. In contrast, Configuration B demonstrates a more progressive damage evolution. The surface-glass-fiber layers act as a sacrificial shielding, suppressing the initial peak force fluctuations and maintaining a higher residual load-carrying capacity. This transition from a sharp drop to a plateau-like response highlights the enhanced damage tolerance afforded by the surface hybridization.

**Figure 9:**
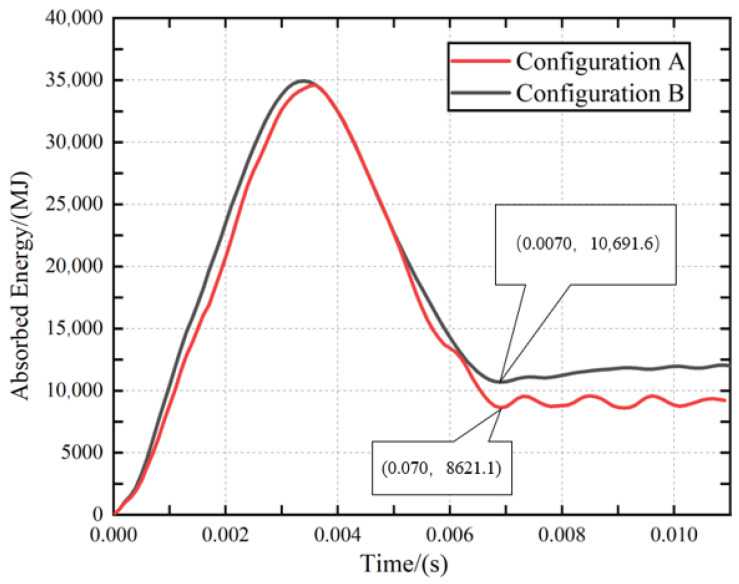
Energy absorption curves for Configuration A and Configuration B.

In this study, the point at the front end of the impact hammer was selected to extract the energy-time history curve. Using an impact energy of 35 J as the total energy, the absorbed energy curve was obtained by subtracting the extracted energy from the total energy, as shown in Figure 9. The energy-time history reveals a distinct difference in the energy dissipation capacity between the two configurations. While both reach a peak energy of approximately 35 J, the permanently absorbed energy for Configuration B is significantly higher than that of Configuration A. This 24% increase in energy absorption is attributed to the synergistic “hybrid effect.” The glass fibers, characterized by a higher failure strain and superior fracture toughness compared to carbon fibers, facilitate more extensive micro-cracking and plastic deformation before final failure. This mechanism effectively redistributes the impact energy, preventing the localized, brittle macro-fracture observed in the pure CFRP baseline. Consequently, the integration of GFRP skins serves as an effective strategy for mitigating impact-induced damage and enhancing the structural integrity of carbon-fiber-based composites.

This improvement arises from the synergistic interaction between carbon and glass fibers. The surface glass fiber layer in Configuration B acts as a protective buffer that attenuates the impact load before it reaches the underlying carbon-fiber plies. Owing to their higher failure strain, glass fibers promote crack deflection and bridging, thereby suppressing rapid crack propagation and maintaining interlaminar integrity. In addition, the mismatch in stiffness and failure strain between the two fiber types induces progressive micro-damage mechanisms, enabling greater energy dissipation through deformation rather than sudden brittle fracture. Consequently, surface hybridization effectively enhances damage tolerance and preserves the overall structural integrity of the composite.

To further validate the proposed impact-resistance mechanisms, the present results are compared with existing studies on carbon/glass hybrid composites. Previous research has shown that glass fibers improve impact performance by enhancing failure strain and promoting progressive damage. Dong and Davies [43] reported increased energy absorption in hybrid laminates, while Çetin [15] attributed improved impact strength to the combination of brittle carbon fiber fracture and ductile glass fiber deformation. These findings are consistent with the present study, where a 24% increase in energy absorption and reduced delamination area are observed in Configuration B.

Moreover, Sözen et al. [16] demonstrated that glass fiber composites exhibit superior impact resistance due to their higher deformability, supporting the buffering role of surface glass fiber layers observed in this work. The crack deflection and branching behavior identified in Configuration B also agree with Shi and Soutis [19], who highlighted the role of heterogeneous interfaces in increasing fracture energy. Unlike previous studies focusing on through-thickness hybridization, this study demonstrates that surface hybridization is particularly effective in mitigating impact damage by modifying stress wave propagation at the contact interface.

## 5. Conclusions

This study conducted a combined experimental and numerical investigation on the low-velocity impact behavior of composite T-stiffened panels with two different configurations. The main conclusions are as follows:

1. Compared with Configuration A, Configuration B exhibited improved damage tolerance under low-velocity impact loading. The introduction of hybrid carbon/glass fiber interfaces modified the crack propagation path, promoting crack deflection and branching, which effectively reduced the overall damage area.
2. The glass fiber layers contributed to enhanced interlaminar integrity through a bridging effect. This effect limited delamination propagation at both 20 J and 35 J impact energies, indicating the beneficial role of localized hybridization in suppressing impact-induced damage.
3. The numerical results showed good agreement with the experimental observations in terms of delamination morphology and damage distribution. This agreement validates the reliability of the proposed damage modeling approach for predicting the impact response of composite T-stiffened panels.
4. In terms of energy absorption, Configuration B achieved a 24% increase in permanent energy absorption at 35 J compared with Configuration A. The hybrid architecture also changed the failure behavior from a relatively brittle damage mode to a more progressive failure response, thereby improving the residual load-bearing capacity.

Overall, this paper demonstrates that localized hybridization using glass fiber layers is an effective strategy for improving the impact resistance and damage tolerance of composite T-stiffened panels. The validated numerical framework can also provide a useful tool for the structural design and optimization of hybrid composite stiffened structures.

## Figures and Tables

**Table 1:** Specimen configuration data.

Configuration	Position	Layup	Number of Layers	Thickness/mm
A	Vertical stiffener-Web	[45/0/−45/0/0/−45/0/45]	8	1.52
	Vertical stiffener-foot	[45/0/−45/0/90/0/−45/0/45]	9	1.71
	Skin-tab	[45/0/−45/90/0/45/−45/−45/45/0/90/−45/0/45]	14	2.66
B	Vertical stiffener-Web	[(0)/45/0/−45/0/0/−45/0/45/(0)]	10	1.62
	Vertical stiffener-foot	[45/0/−45/0/90/0/−45/0/45]	9	1.71
	Skin-tab	[45/0/−45/90/0/45/−45/−45/45/0/90/−45/0/45]	14	2.66

**Table 2:** Impact energy and results.

Number	Configuration	Impact Energy (J)	Damage Area	Damage to the Appearance
1	A	35	flange: 52 mm × 14 mm; web: 40 mm × 27 mm.	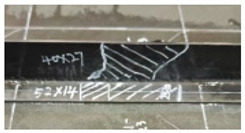
2		20	flange: 60 mm × 8 mm; web: 45 mm × 26 mm.	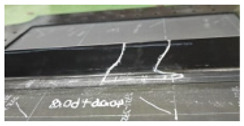
3	B	35	flange: 51 mm × 11 mm; web: 33 mm × 27 mm.	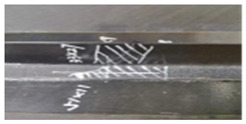
4		20	flange: 7 × 8 mm; 25 mm × 9 mm; web: 38 mm × 27 mm.	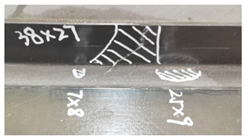

**Table 3:** Comparison of experimental damage areas for Configuration A and Configuration B under different impact energies.

Configuration	Impact Energy (J)	Experimental (mm^2^)	Area Reduction (%)
A	35	1419.28	
B	35	1139.82	19.69%
A	20	1295.25	
B	20	805.41	37.81%

**Table 4:** Comparison of simulated damage areas for Configuration A and Configuration B under different impact energies.

Configuration	Impact Energy (J)	Simulated (mm^2^)	Area Reduction (%)
A	35	1712.46	
B	35	1305.18	23.78%
A	20	1048.73	
B	20	731.44	30.25%

## Data Availability

The original contributions presented in this study are included in the article. Further inquiries can be directed to the corresponding authors.
